# Genome-wide association study and polygenic score assessment of insulin resistance

**DOI:** 10.3389/fendo.2024.1384103

**Published:** 2024-06-13

**Authors:** Usama Aliyu, Umm-Kulthum Ismail Umlai, Salman M. Toor, Asma A. Elashi, Yasser A. Al-Sarraj, Abdul Badi Abou−Samra, Karsten Suhre, Omar M. E. Albagha

**Affiliations:** ^1^ College of Health and Life Sciences (CHLS), Hamad Bin Khalifa University (HBKU), Qatar Foundation (QF), Doha, Qatar; ^2^ Qatar Genome Program (QGP), Qatar Foundation Research, Development and Innovation, Qatar Foundation (QF), Doha, Qatar; ^3^ Qatar Metabolic Institute, Hamad Medical Corporation, Doha, Qatar; ^4^ Bioinformatics Core, Weill Cornell Medicine-Qatar, Doha, Qatar; ^5^ Department of Biophysics and Physiology, Weill Cornell Medicine, New York, NY, United States

**Keywords:** insulin resistance, beta cell, type 2 diabetes, GWAS, polygenic score

## Abstract

Insulin resistance (IR) and beta cell dysfunction are the major drivers of type 2 diabetes (T2D). Genome-Wide Association Studies (GWAS) on IR have been predominantly conducted in European populations, while Middle Eastern populations remain largely underrepresented. We conducted a GWAS on the indices of IR (HOMA2-IR) and beta cell function (HOMA2-%B) in 6,217 non-diabetic individuals from the Qatar Biobank (QBB; Discovery cohort; n = 2170, Replication cohort; n = 4047) with and without body mass index (BMI) adjustment. We also developed polygenic scores (PGS) for HOMA2-IR and compared their performance with a previously derived PGS for HOMA-IR (PGS003470). We replicated 11 loci that have been previously associated with HOMA-IR and 24 loci that have been associated with HOMA-%B, at nominal statistical significance. We also identified a novel locus associated with beta cell function near *VEGFC* gene, tagged by rs61552983 (*P* = 4.38 × 10^-8^). Moreover, our best performing PGS (Q-PGS4; Adj R^2^ = 0.233 ± 0.014; *P* = 1.55 x 10^-3^) performed better than PGS003470 (Adj R^2^ = 0.194 ± 0.014; *P* = 5.45 x 10^-2^) in predicting HOMA2-IR in our dataset. This is the first GWAS on HOMA2 and the first GWAS conducted in the Middle East focusing on IR and beta cell function. Herein, we report a novel locus in *VEGFC* that is implicated in beta cell dysfunction. Inclusion of under-represented populations in GWAS has potentials to provide important insights into the genetic architecture of IR and beta cell function.

## 1 Introduction

Type 2 diabetes (T2D) is a metabolic disorder that poses an alarming health concern worldwide and greatly affects quality of life, healthcare and mortality (1). The burden of T2D is on the rise globally and the International Diabetes Federation (IDF) estimated that more than 463 million people, corresponding to about 6.28% of the global population are affected by T2D in 2019 (2). In the Middle East and North African (MENA) region, about 4.8 million (12.8% of the region population) adults are affected by T2D, and the figure is projected to reach 700 million globally and 25.2 million in MENA by 2045 (2, 3). Moreover, the prevalence of T2D in Qatar has reached ~20% of the population (4), and was estimated to account for ~7% of the total disease burden and ~10% of total mortality in Qatar in 2020 (5).

Insulin resistance (IR) and beta cell dysfunction are considered as the major drivers in the pathogenesis of T2D. IR is defined by the gradual diminished ability of insulin to adequately induce cellular response for glucose uptake and utilization (6) and is caused by the interplay of a multitude of factors including lifestyle and genetics. It is also increasingly becoming evident that genetic susceptibility is an important risk factor for developing IR (7, 8). Scientists have leveraged the advent of Genome-Wide Association Studies (GWAS) and Next-Generation Sequencing (NGS) technologies to identify common and rare genetic variants associated with various diseases and traits, including T2D and IR. To date, ~24 genetic variants in 11 independent loci have been associated with IR, as measured by the Homeostasis Model Assessment-Insulin Resistance (HOMA-IR) method, while ~145 variants in 24 loci have been associated with beta cell function, as measured by HOMA-%B (9, 10). However, majority of these studies are largely dominated by populations of European and Asian descent, with little representation from Middle Eastern populations. Studies have shown that trait-associated variants have different allele frequency and effect sizes across populations, which complicates the development and utility of European-based polygenic scores (PGS) when applied on other populations (11). GWAS in Middle Eastern populations are warranted to bridge the gap and to counter the bias in available genomic literature and data.

Medication and intensive lifestyle interventions have been shown to prevent T2D progression (12). Identification of common genetic variants that increase susceptibility to IR and beta cell dysfunction has merits in ascertaining individuals at higher genetic risk of developing T2D for early intervention. Recently Khera et al. (2018) leveraged results from GWAS to identify individuals at greater than threefold increased risk of developing T2D caused by common variants as compared to the risk conferred by monogenic mutations (13). However, common genetic variants associated with IR and beta cell function in Middle Eastern populations have not been previously investigated. Herein, we performed the first comprehensive GWAS on IR and beta cell function using whole-genome sequencing data from population-based cohort of Qatar biobank (QBB; n = 6,217). Moreover, we developed the first polygenic score (PGS) for standard measure of IR in the Qatari population. Overall, our study highlights the genetic architecture of IR and beta cell function in underrepresented populations.

## 2 Methods

### 2.1 Study subjects and clinical characteristics

This study was conducted on participants from QBB, a population-based prospective initiative by Qatar Foundation to promote biomedical research in Qatar and worldwide. QBB recruits adults (aged ≥ 18 years) who are permanent or long-term (≥ 15 years) residents of Qatar and covers extensive baseline social, demographic, clinical, metabolic and behavioral phenotypic data, in addition to collecting biological samples (14). The present study was restricted to include Qatari subjects only.

The study was conducted under ethical approvals from the Institutional Review Boards of QBB (Approval No. E/2017/QGP -RES-PUB-009/0015) and Hamad Bin Khalifa University (Approval No. QBRI-IRB 2021-03-078). All participants provided written informed consent prior to participation in the study.

### 2.2 Quantitative traits measurements

All QBB participants attended assessment and interview sessions with healthcare professionals and filled out standardized questionnaires. The questionnaires collected information on participants’ current and past health conditions, smoking history, occupation, sociodemographic, physical activity, and lifestyle. Moreover, information on prevalent health condition, family history and medication use was also collected. Study participants also provided biological samples including blood, urine and saliva samples of which part was transferred to the College of American Pathologists (CAP) accredited diagnostic laboratories of Hamad General Hospital for measurements of clinical biomarkers, while Whole Genome Sequencing was conducted by the Qatar Genome Program (QGP). Serum C-peptide levels were measured using the sandwich electrochemiluminescence immunoassay using Elecsys C-Peptide kit (Roche, Basel, Switzerland), while fasting glucose levels in serum were measured using the enzymatic method with GLUC3 glucose hexokinase kit (Roche) on a COBAS instrument (Roche).

### 2.3 Phenotype definition

Homeostasis Model Assessment 2 (HOMA2) for insulin resistance (HOMA2-IR), beta cell function (HOMA2-%B) and insulin sensitivity (HOMA2-%S) were calculated for each subject with fasting (≥8 hours) levels of glucose and C-peptide using HOMA2 calculator (https://www.dtu.ox.ac.uk/homacalculator/). All traits were normalized prior to performing the GWAS, using rank-based inverse normal transformation in R (ver. 3.4.0).

### 2.4 Subject inclusion criteria

QBB participants were categorized as Type 1 diabetes (T1D) if they were exclusively receiving insulin and their serum C-peptide level was < 0.5ng/ml. Type 2 diabetes, if they were not classified as T1D and declared they have diabetes, or are on diabetes treatment. Newly diagnosed diabetes, if their HbA1C > 6.5 and/or random glucose level > 11.1 mmol/l (>200 mg/dl) and did not self-report as having diabetes. Subjects were otherwise classified as non-diabetes if they did not fall in any of the aforementioned categories. Subjects without diabetes and who were informative for fasting glucose, insulin, C-peptide levels, and genotype data were used in this study comprising 2,170 subjects from the first QBB data release (Discovery cohort) and 4,047 subjects from the second QBB data release (Replication cohort).

### 2.5 Whole genome sequencing

The DNA extraction and whole genome sequencing protocols have been previously described (11). Briefly, extracted DNA was used to construct Genomic libraries and sequenced on HiSeq X Ten (illumina, USA) with a minimum average coverage of 30x and DNA sequencing was conducted at the sequencing facility of Sidra Medicine, Doha, Qatar. Reads were aligned to GRCh38 reference genome using bwa.kit (v0.7.12). The variants were jointly called to generate all gVCF files at once following the GATK 3.4 best practices. Stringent quality control (QC) measures were applied to exclude low quality genotypes and samples using the whole-genome association analysis toolset, PLINK (ver. 2.0) (15). At the genotype level, SNPs with minor allele frequency (MAF) < 1%, genotype call rate < 90%, Hardy-Weinberg *P* value < 1×10^−6^, and those on X chromosome were removed. A total of 8,262,420 QC-passed variants were used in downstream analysis. At the sample level, excessively heterozygous, gender ambiguous, call rate < 95% and duplicate samples were also removed. Ancestry outliers were identified using the multidimensional scaling (MDS) function in PLINK 2.0 (15). Using a Linkage Disequilibrium (LD) cut-off of *r^2^ =* 0.05 in a window of 200 independently pruned set of SNPs, pairwise identity by-state (IBS) matrix was generated. Subjects that deviate ±4 SD from the mean are considered as population outliers and thus removed.

### 2.6 Genome-wide association analysis

The association was tested between the HOMA2-IR and HOMA2-%B values and the genotypes of study subjects using the R package; SAIGE (Scalable and Accurate Implementation of GEneralized mixed model). SAIGE uses optimization strategies to correct for relatedness in addition to reduced computational cost (16). The association test was done in two models: without (Model A) and with (Model B) adjusting for BMI in addition to adjusting for age, sex, and the first ten genetic principal components (PC1 to PC10).

GWAS was performed using a two-stage approach; a discovery stage based on 2,170 subjects from the first QBB data release. SNPs that reached suggestive significance of *P* < 5 × 10^-5^ in the discovery were carried forward for validation in 4,047 subjects from the second QBB data release (replication set). The results of the association from the discovery and replication stages were combined in a fixed-effect and random-effect meta-analysis using PLINK 2.0 (15). A *P*-value of < 5 × 10^-8^ was considered genome-wide significant. Cochran’s *Q* was used to assess the heterogeneity across the two analyses (17). The effect of the associated genotypes was determined by the size and direction of the beta-value. Manhattan and quantile–quantile plots were generated to visualize association results using the R package qqman (18). SNPs were considered as novel variants when no previous reports are found in various genomic databases; including GWAS catalog (9), Phenoscanner (10), Hugeamp (19) and NCBI database (20). Loci are considered novel when no SNP around 250 kb window of the identified SNP has been previously reported. Our discovery dataset was adequately powered (95%) to detect variants with an effect size (beta) of 0.175 at genome-wide significance (*P*<5 x 10^-8^).

### 2.7 Comparison with other populations

We relied on studies conducted on HOMA1 due to lack of previous GWAS of HOMA2. The previously reported SNPs associated with any of the two traits (HOMA-IR and HOMA-%B) were checked for replication in the discovery GWAS at nominal significance (*P*<0.05). In the event where exact SNP was not replicated, we searched for evidence of loci replication; we searched for SNPs with nominal evidence of association (*P* < 0.05) within 250 kb window of the reported SNP. The effect allele frequency of the lead SNP was compared between our study (QBB) and Europeans (EUR), East Asians (EAS), South Asians (SAS), Africans (AFR), and Admixed Americans (AMR) from the 1000 Genome Project (21).

### 2.8 Polygenic score derivation and optimization

Polygenic score (PGS) is a quantitative metric that informs about an individual’s genetic susceptibility to a certain disease or trait based on the cumulative effect of alleles associated with the trait. We used our discovery GWAS summary statistics of IR (HOMA2-IR) to derive PGS using PLINK ver.1.9 software (15), based on Clumping and Thresholding (C + T) method. C + T relies on linkage disequilibrium (LD) to clump SNPs with LD (r^2^) and association *P*-values using different set of thresholds. Each clump consists of an index SNP independent of the other clumps based on the pre-defined LD (r^2^) threshold. Each set of independent SNPs identified by this method was used as a PGS panel referred to as QBB-derived PGS and numbered from Q-PGS1 to QGP-6, based on decreasing *P*-value. Several *P*-value thresholds were tested to identify the optimal PGS from BMI-adjusted (Model B) GWAS summary statistics. PGS were derived over a range of *P*-values (5.0 × 10^-1^ to 5.0 × 10^-6^) with r^2^ = 0.2. A total of 6 Q-PGS panels were developed. To identify the optimal PGS, the scores were tested in the second data release of QBB comprising 4,047 individuals from the replication dataset. Linear regression was carried out to assess the performance of PGS, adjusting for gender, age, BMI and first 10 PCs as predictor variables in the model. The PGS with the best predictive capacity was determined based on maximal adjusted R^2^ values and least number of SNPs. Correlation between PGS and normalized HOMA2-IR values was also assessed using Spearman’s Rank Correlation coefficient (Rho). Moreover, we generated a quantile plot for the top-performing Q-PGS and investigated association with HOMA2-IR values. Lastly, we assessed the performance of a previously developed PGS for IR (PGS003470; https://www.pgscatalog.org/score/PGS003470/) by Zhang et al., from European populations (22), when applied to the Qatari population (QBB).

## 3 Results

### 3.1 Characteristics of study participants

**Figure 1:**
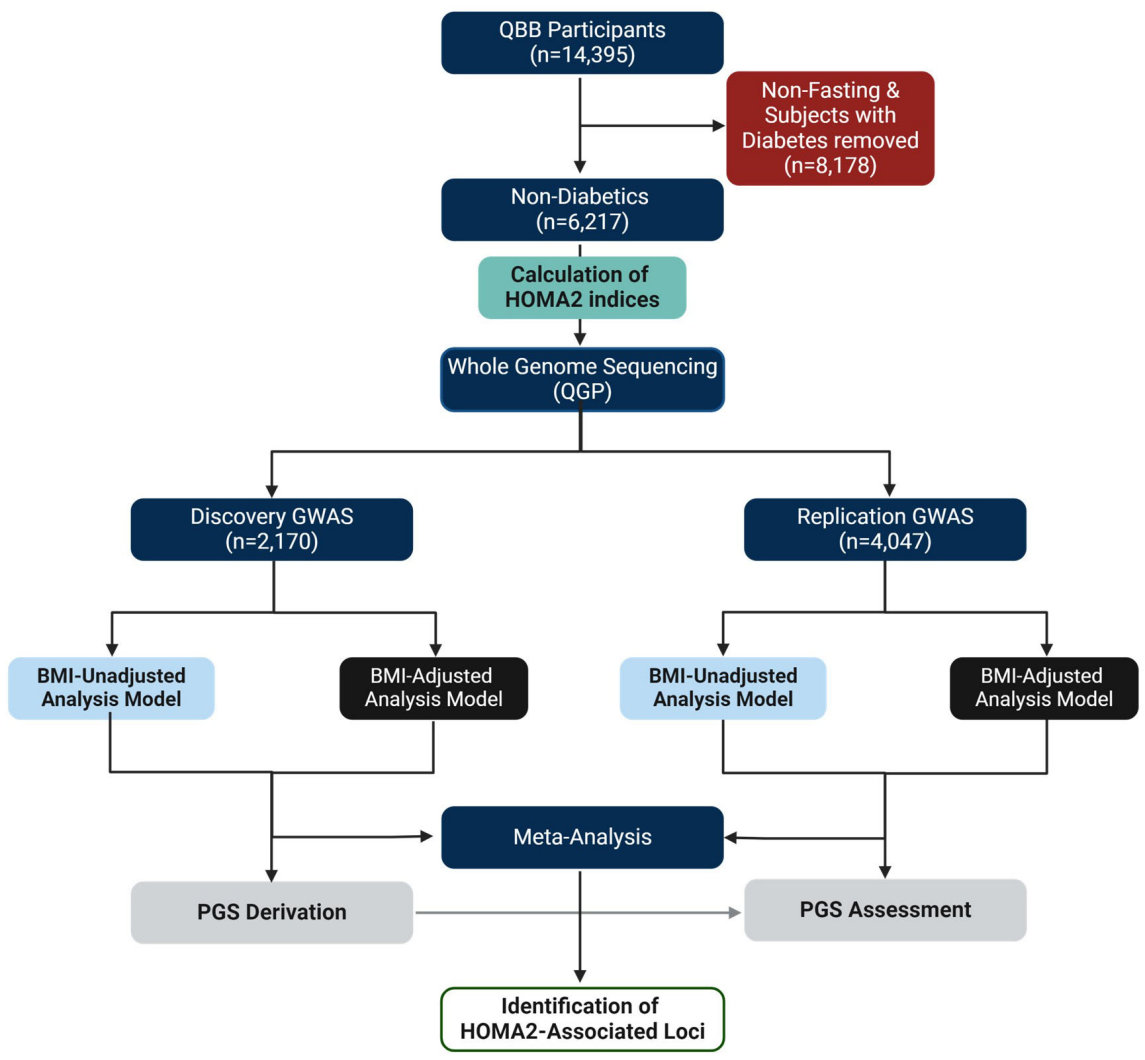
Study Design. This study was based on the Qatar Biobank (QBB) participants (n = 14,395). Subjects with diabetes and those without fasting measurements (n = 8,178) were removed. The GWAS cohort included only subjects without diabetes with fasting levels of glucose and C-peptide (n = 6,217). The phenotype (HOMA2-IR, HOMA2-%B, HOMA2-%S) values were calculated using HOMA2 calculator. Whole genome sequencing (WGS) data was provided by the Qatar Genome Program (QGP). The GWAS was conducted in discovery (n = 2,170) and replication (n = 4,047) using SAIGE and meta-analyzed using Plink in BMI adjusted and BMI-unadjusted models. Polygenic scores (PGS) were derived from discovery dataset and tested in the replication dataset.

The overall study design is depicted in **Figure 1** and the clinical characteristics of the study subjects (*n* = 6,217) are listed in **Table 1**. The average age was 37.7 years with a mean HOMA2-IR of 1.52, mean HOMA2-%B of 125.8 and mean HOMA2-%S of 78.2. Subjects were also characterized with a mean BMI of 29.1, while ~75% were obese or overweight. The gender distribution was 41.1% (2,558) males and 58.9% (3,659) females.

**Table 1:** Characteristics of study subjects.

Characteristics	Discovery (*n* = 2,170)	Replication (*n* = 4,047)	All (*n* = 6,217)
Male n (%)	840 (38.7)	1,718 (42.5)	2,558 (41.1)
Female n (%)	1,330 (61.3)	2,329 (57.5)	3,659 (58.9)
Mean age (years)	37.5 ± 11.8	37.8 ± 12.1	37.7 ± 12.0
C-peptide (nmol/L)	0.66 ± 0.30	0.72 ± 0.31	0.70 ± 0.30
Glucose (mmol/L)	5.17 ± 0.57	4.86 ± 0.55	4.97 ± 0.58
HOMA2-IR	1.47 ± 0.68	1.57 ± 0.69	1.52 ± 0.69
HOMA2-%B	114.2 ± 30.3	137.3 ± 39.1	125.8 ± 34.7
HOMA2-%S	80.9 ± 34.0	75.5 ± 31.7	78.2 ± 32.9
BMI (kg/m^2^) *	29.1 ± 5.96	29.0 ± 5.96	29.1 ± 5.96
Obese	868 (40.0)	1,603 (39.6)	2,471 (39.7)
Overweight	758 (34.9)	1,440 (35.6)	2,198 (35.4)
Normal weight	496 (22.8)	916 (22.6)	1,412 (22.7)
Underweight	49 (2.3)	87 (2.2)	136 (2.2)

Characteristics of the QBB cohort batch 1 (Discovery) and batch 2 (Replication). Quantitative variables are expressed as mean ± standard deviation, categorical variables are expressed as number (percentage). HOMA2-IR: Homeostasis Model Assessment 2 – Insulin Resistance, HOMA2-%B: Homeostasis Model Assessment 2- Beta cell function, HOMA2-%S: Homeostasis Model Assessment 2- Insulin Sensitivity. *: Subjects are classified as obese if their BMI (≥ 30 *kg/m* ^2^), overweight (24.9 < BMI < 30 *kg/m* ^2^), normal (18.5 ≤ BMI ≤ 24.9 *kg/m* ^2^), underweight (BMI < 18.5 *kg/m* ^2^).

### 3.2 GWAS of HOMA2-IR

**Figure 2:**
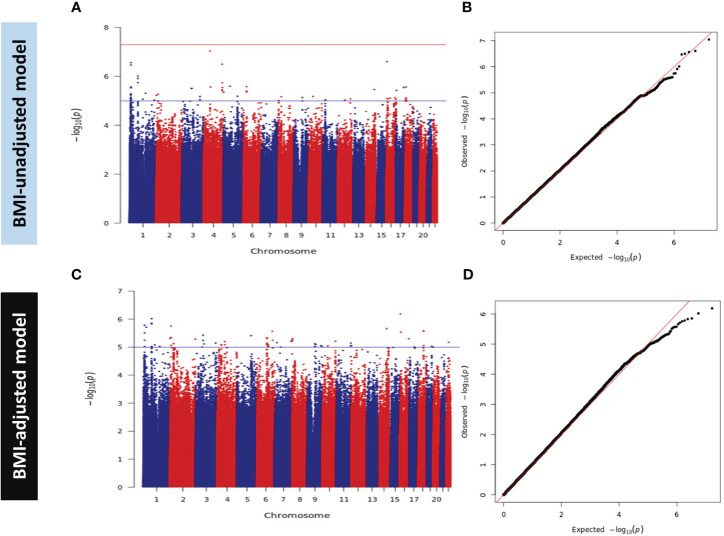
Manhattan and Q-Q Plots for HOMA2-IR in Discovery GWAS. **(A)** Manhattan plot and **(B)** Quantile-Quantile (Q-Q) plot of discovery association results in the BMI-unadjusted model (Model A). **(C)** Manhattan plot and **(D)** Q-Q plot of discovery GWAS in BMI-adjusted model (Model B). Manhattan plots represent the -log10 *P* (significance) on y-axis for SNPs represented on the x-axis based on their chromosomal position. The blue horizontal line represents suggestive evidence of association (*P* < 5 × 10^-5^). The red horizontal line represents the genome-wide significance threshold (*P* < 5 × 10^-8^). Q-Q plots represent the quantile distribution of observed p-values versus the expected p-values for all SNPs.

A total of 8,262,420 SNPs were tested for associations with IR (HOMA2-IR) in the discovery stage in non-BMI and BMI-adjusted models, referred to as models A and B, respectively. The GWAS results are presented as Manhattan and Quantile-Quantile (Q-Q) plots (**Figure 2**). There was no evidence of genomic inflation since the genomic inflation factor (λ_GC_) was 1.0.

We did not detect any genome-wide significant variants (*P* < 5.0 × 10^-8^) in the discovery dataset. However, 456 and 558 SNPs showed suggestive evidence of association with a *P* value < 5 × 10^-5^ in models A and B, respectively. These SNPs were carried forward for validation in subjects from the replication set of QBB (*n* = 4,047) using the same model of regression and adjustment. Meta-analysis of the two studies (discovery and replication) did not reveal any GWAS significant signal (**Supplementary Figures 2A, B**). Of note, SNPs rs61552983 (4q34.3) near Vascular Endothelial Growth Factor C (*VEGFC)* in chromosome 4 and rs6912701 (6p21.32) near *HLA-DRA* in chromosome 6 attained a *P* value of < 5 × 10^-6^ in model A. Moreover, SNPs in chromosome 4 tagged by rs13105357 (4q35.1) near *CDKN2AIP* and rs9839000 (3q27.1) in chromosome 3 near *FAM131A* also showed suggestive evidence of association with a *P* value < 5 × 10^-6^ in model B (data not shown).

At the time of writing, 24 SNPs in 11 independent loci were reported to be associated with HOMA-IR at the genome-wide significance level (*P* < 5.0 × 10^-8^) in the GWAS catalog (9), Phenoscanner (10) and published literature. However, no GWAS have been previously conducted on HOMA2 indices. Despite the disparities between HOMA2 and the conventional HOMA, we replicated 3 exact SNPs in 3 loci in model A and 4 exact SNPs in 3 loci in model B (**Supplementary Table 1**) at nominal significance threshold (*P* < 0.05), all with consistent direction of effect to those previously reported. For the other 8 loci that did not show nominal replication for the exact SNP (*P* > 0.05), all contained signals within ±250 kb with evidence for nominal replication (*P* < 0.05). We reported the lead SNP in QBB within ±250 kb of the previously reported SNPs, number of SNPs within the window and compared their allele frequency between QBB and the 5 super populations in the 1000 genome project (**Supplementary Tables 2, 3**). Correlations of the allele frequency in both models showed highest correlations with Europeans (R^2^ = 0.97 in model A, and R^2^ = 0.92 in model B) compared to other populations ancestries in the 1000 genome project (**Supplementary Figure 1**).

### 3.3 GWAS of HOMA2-%B

**Figure 3:**
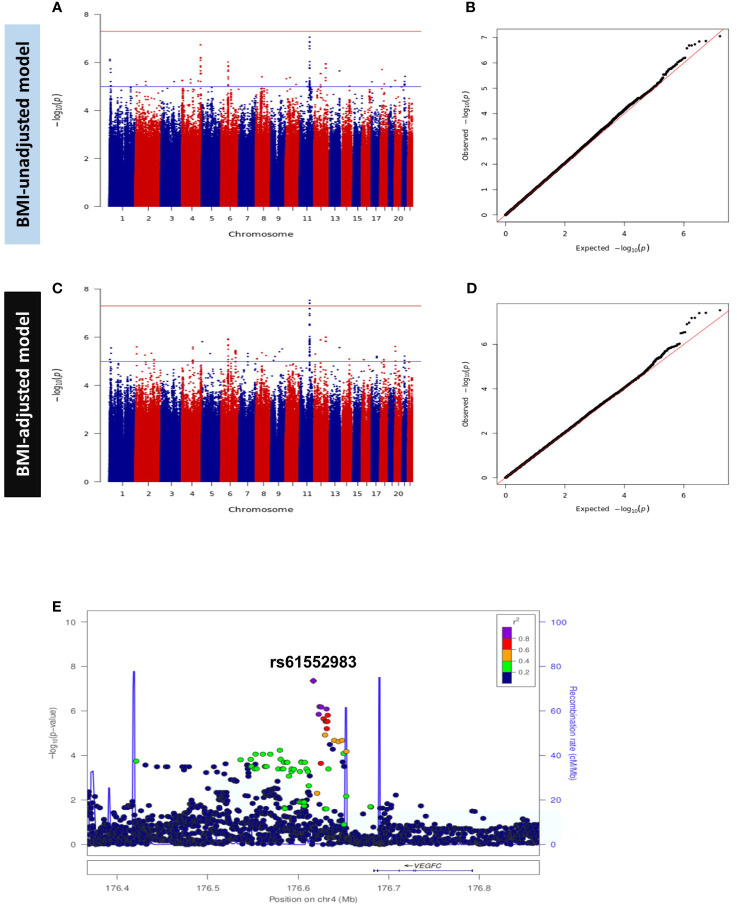
Manhattan and Q-Q Plots for HOMA2-%B in Discovery GWAS. **(A)** Manhattan plot and **(B)** Quantile-Quantile (Q-Q) plot of discovery association results in the BMI-unadjusted model (Model A). **(C)** Manhattan plot and **(D)** Q-Q plot of discovery GWAS in BMI-adjusted model (Model B). Manhattan plots represent the -log10 *P* (significance) on y-axis for SNPs represented on the x-axis based on their chromosomal position. The blue horizontal line represents suggestive evidence of association (*P* < 5 × 10^-5^). The red horizontal line represents the genome-wide significance threshold (*P* < 5 × 10^-8^). Q-Q plots represent the quantile distribution of observed p-values versus the expected p-values for all SNPs. **(E)** Regional association plot of the novel locus (tagged by rs61552983) associated with beta cell function (HOMA2-%B). SNPs are plotted with meta-analysis p-values (-log10) as a function of genomic position.

We performed GWAS for beta cell function (HOMA2-%B) in the discovery dataset in models A and B. Both models were adjusted for age, sex and the first ten principal components (PC1 – PC10) and showed no evidence of genomic inflation (λ_GC_ = 1.0). The GWAS results are presented as Manhattan and Q-Q plots (**Figure 3**). After meta-analysis, 2 independent loci; a novel locus on 4q34.3 and a previously reported locus on 11q14.3 showed genome-wide significant association with HOMA2-%B in model A and only 11q14.3 in model B (**Table 2**, **Supplementary Figures 2C, D**). SNPs at chromosome 4q34.3 (lead SNP: rs61552983, *P*-value: 4.38 × 10^-8^) near *VEGFC* were significantly associated with HOMA2-%B in Model A (**Figure 3E**). Importantly, the locus has not been previously reported to be associated with beta cell function or any glycemic traits. However, the association *P*-value changed from 4.38 × 10^-8^ to 3.60 × 10^-6^ after adjusting for BMI (Model B). In contrast, SNPs at chromosome 11q14.3 (lead SNP: rs10830963) near *MTNR1B* were significantly associated with HOMA2-%B in both models A and B. Interestingly, the strength of the association increased from 2.07 × 10^-14^ to 2.74 × 10^-16^ after adjusting for BMI (Model B).

**Table 2:** HOMA2-%B-associated loci in QBB at *P* < 5 x 10^−8^.

							Discovery		Replication		Meta-analysis			
Chr	Locus	No. SNPs	Lead SNP	BP Position	Allele (Effect/ Other)	EAF	Effect size (Beta)	*P* value	Effect size (Beta)	*P* value	Effect size (Beta)	*P* value	*Q*	Nearest coding gene
**4**	**4q34.3**	**19**	**rs61552983**	**176616690**	**G/A**	**0.95**	**-0.346**	**1.85x10^-7^**	**-0.157**	**6.40x10^-3^**	**-0.238**	**4.38x10^-8^**	**0.031**	***VEGFC***
**4**	**4q34.3**	**12**	rs61552983^*^				**-0.260**	**3.48x10^-5^**	**-0.139**	**1.11 x10^-2^**	**-0.191**	**3.60x10^-6^**	**0.15**	
**11**	11q14.3	31	rs10830963	92975544	C/G	0.72	+0.176	2.05x10^-7^	+0.143	1.47x10^-8^	+0.155	2.07x10^-14^	0.44	*MTNR1B*
**11**	11q14.3	45	rs10830963^*^				+0.175	3.93x10^-8^	+0.147	1.01x10^-9^	+0.157	2.74x10^-16^	0.48	

Chr, Chromosome; BP, Base pair; EAF, Effect Allele Frequency; Q, Cochran’s Q value. Bold text indicates novel variant in a novel locus. *Model B (After BMI-adjustment).

**Figure 4:**
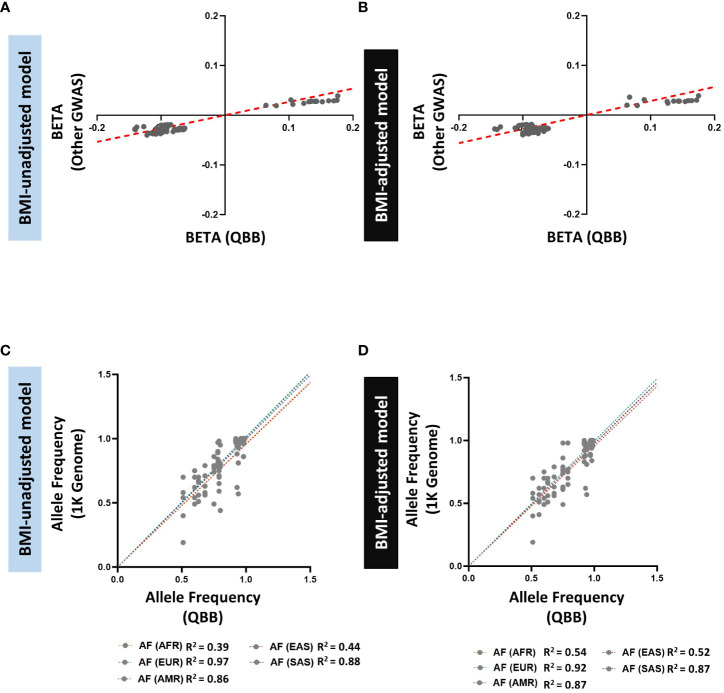
Comparison of allele frequencies and effect sizes (BETA) of HOMA2-%B-replicated loci identified in the GWAS catalog and QBB Cohort. **(A, B)** Correlation of effect sizes (beta) for replicated loci between QBB and GWAS catalog in **(A)** BMI-unadjusted (R2=0.92) and **(B)** BMI-adjusted (R2=0.85) models. **(C, D)** Correlation of the allele frequency of the lead SNPs in QBB within ±250 kb of previously reported SNPs in (C) BMI-unadjusted and **(D)** BMI-adjusted models between QBB and European (EUR), African (AFR), East Asian (EAS), South Asian (SAS) and Admixed American (AMR) ancestry subjects from the 1000 Genome project.

We also searched for evidence of replication of the previously reported 145 SNPs in 21 loci associated with HOMA-%B. We successfully replicated 91 exact SNPs in 5 loci in model A and replicated 98 exact SNPs in 5 loci in model B (**Supplementary Table 4**) at a nominal significance threshold (*P* < 0.05) with consistent effect direction. Ninety replicated SNPs are common to both models and we observed a strong correlation of effect size and direction (R^2^ = 0.92 in model A and R^2^ = 0.85 in model B) between QBB and other GWAS (**Figures 4A, B**). For the other 16 loci that did not show nominal replication for the exact SNP (*P* > 0.05), they all contained signals within ±250 kb with evidence for nominal replication (*P* < 0.05). We reported the lead SNP in QBB within ±250 kb of the previously reported SNPs in both models, the number of SNPs within the window and compared their allele frequency between QBB and the 5 super populations in the 1000 genome project (**Supplementary Tables 5, 6**). As observed in HOMA2-IR, comparison of the allele frequency of the lead SNP in the loci showed the highest correlation with the European (R^2^ = 0.97 in model A and R^2^ = 0.92 in model B) and QBB compared to other populations ancestries in the 1000 genome project (**Figures 4C, D**).

### 3.4 Developing and optimizing polygenic scores

**Figure 5:**
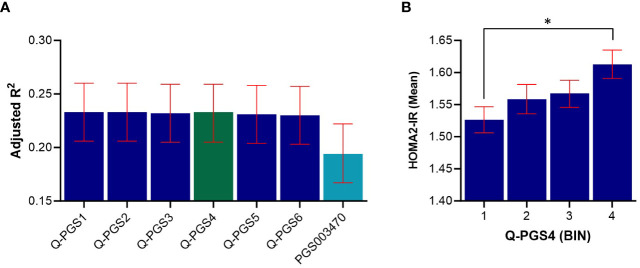
Predictive performance assessment of Q-PGS for insulin resistance. **(A)** Bar chart shows the adjusted R^2^ values of the 6 Q-PGS. Analyses were adjusted for age, sex, BMI and PCs1-10. **(B)** Quantile bar chart shows the mean HOMA2-IR values for each score bin for Q-PGS4; bins were divided into four equal groups of participants scores (n= ~1,102 in each quantile). Asterisk (*) represents statistically significant (*P* < 0.05). Error bars represent the standard error. Q-PGS, QBB-derived Polygenic Scores.

We developed 6 PGS for IR following clumping and thresholding (C + T) method, as described in the methods section. The performance of the PGS varied depending on the *P*-value thresholds used for selecting the genetic variants. The developed Q-PGS are listed in **Table 3**. Among the Q-PGS, the top 3 performing scores were Q-PGS1 (Adj. R^2^ = 0.233; *P* = 3.79x10^-5^), Q-PGS2 (Adj-R^2^ = 0.233; *P* = 1.20x10^-4^) and Q-PGS4 (Adj. R^2^ = 0.233; *P* = 1.55x10^-3^) in the linear regression model (**Figure 5A**). To determine the optimal PGS we scrutinized the panel sizes and there was a substantial difference in the number of SNPs included in the 3 PGS; a 300-fold difference from 1,390 to 480,757 SNPs; Q-PGS4 was selected as the optimal PGS based on the largest Adj-R^2^ and smaller number of SNPs (1,390).

**Table 3:** Candidate polygenic scores (PGS) for insulin resistance (IR).

PGS Score	PGS Name	Available variants/Variants in score (%)	Correlation			
			Adjusted R^2^ (95% CI)	SE	*P* value	Rho
*P*<5x10^-1^_r^2^<0.2	Q-PGS1	480,757/488,609 (98.4%)	0.233 (0.206-0.260)	0.0138	3.79x10^-5^	0.0670
*P*<5x10^-2^_r^2^<0.2	Q-PGS2	76,963/78,116 (98.5%)	0.233 (0.206-0.260)	0.0138	1.20x10^-4^	0.0657
*P*<5x10^-3^_r^2^<0.2	Q-PGS3	10,598/10,749 (98.6%)	0.232 (0.205-0.259)	0.0138	4.95x10^-4^	0.0563
*P*<5x10^-4^_r^2^<0.2	Q-PGS4	1,390/1,414 (98.3%)	0.233 (0.205-0.259)	0.0138	1.55x10^-3^	0.0356
*P*<5x10^-5^_r^2^<0.2	Q-PGS5	183/187 (97.9%)	0.231 (0.204-0.258)	0.0138	4.40x10^-2^	0.0351
*P*<5x10^-6^_r^2^<0.2	Q-PGS6	12/13 (92.3%)	0.230 (0.203-0.257)	0.0138	6.58x10^-1^	0.0095
PGS003470	PGS003470	657,593/775,999 (84.7%)	0.194 (0.167-0.222)	0.0141	5.45x10^-2^	0.0265

PGS, Polygenic Score; SE, Standard Error; CI, Confidence interval; Rho, Spearman correlation coefficient.

Next, we evaluated the performance of a previously derived PGS for HOMA-IR (PGS003470) when applied on the QBB cohort. PGS003470 was developed by Zhang et al. (22), for IR using LDpred2 algorithm and is currently the only available PGS for HOMA-IR in the PGS catalog. Our top QBB-derived PGS (Q-PGS4) performed better than PGS003470 with an adjusted-R^2^ of 0.233 compared to 0.194 (**Figure 5A** and **Table 3**). In addition, the quantile distribution for Q-PGS4 also showed steady increase with HOMA2-IR values (**Figure 5B**). Furthermore, there was a significant difference (*P <*0.05) between the highest and lowest PGS quantiles.

## 4 Discussion

We conducted a comprehensive GWAS to identify the genetic determinants of the two major drivers of T2D: IR and beta cell dysfunction. We utilized deep phenotypic data provided by QBB and WGS data by QGP that provides complete genomic coverage, as opposed to the commonly used targeted SNP arrays for GWAS that suffer from imputation errors and bias (23). Although previous studies used HOMA1, we employed the use of HOMA-2 as it reflects the physiological insulin hemostasis and accommodates modern insulin assays (24). In addition, although HOMA2 indices can also be calculated from fasting insulin levels instead of C-peptide, insulin concentration may be partly affected by the hepatic metabolism (25). Since C-peptide is more stable than insulin and is released in equimolar concentration with insulin, it seemed to reflect better the actual index and was thus used in the calculation. Of note, the mean beta cell function (HOMA2-%B = 125.8%) was elevated in our cohort which could be attributed to commensurate compensatory mechanisms of beta cells against reduced insulin sensitivity (HOMA2-%S = 78.2%) (26). The high prevalence of obesity in our cohort also support involvement of augmented beta cell activity to counter insulin sensitivity but requires further investigations to confirm.

We did not identify any genome-wide significant association with IR (HOMA2-IR). This may be due, at least in part, to the limited power of the sample size to detect associations. Moreover, there were only a handful of variants reported to be significantly associated with IR in larger studies as opposed to beta cell function (27). However, among other loci that showed suggestive evidence of association is the 4q34.3 near *VEGFC*. Previous studies have shown that circulating VEGFC levels correlate with metabolic and lipid parameters (28) and are elevated in obese patients (28, 29). Moreover, transgenic overexpression of *VEGFC* has been shown to induce weight gain and IR in mice (30), while variants in *VEGFC* have been associated with lipid traits such as Waist-to-Hip (WHR) ratio (31). However, *VEGFC* has not been directly associated with IR or beta cell function in humans. We replicated a consistent signal for IR (rs780094 near *GCKR*) (27, 32) with consistent direction of effect and allele frequency, after BMI adjustment. We also observed evidence of replication of all previously reported loci associated with HOMA-IR by replicating SNPs at nominal significance (*P*<0.05) within 250kb window of previously reported SNPs. However, some of the replicated SNPs are not in strong LD with the reported SNP suggesting locus heterogeneity. The replication of all previously known loci also suggests that although HOMA-IR and HOMA2-IR are calculated in slightly different ways, they are indexing common trait. The comparison of the allele frequency of the lead SNP between the QBB and the 1000 genome super populations showed higher correlations with the European populations, which is consistent with previous reports in which the allele frequency of QBB variants was compared with European, African and Japanese populations (11).

For beta cell function, we identified 2 variants that reached genome-wide significant association. The SNP rs10830963 in the intronic region of Melatonin Receptor 1B (*MTNR1B*) has been consistently and robustly associated with beta cell function (27, 33). The increase in the strength of association of this SNP from 2.07 × 10^-14^ to 2.74 × 10^-16^ after adjusting for BMI further affirms its association with beta cell function. *MTNR1B* is a protein coding gene that has been associated with type 2 diabetes in several studies (34–37) and glycemic traits (36, 38, 39). *MTNR1B* encodes for melatonin receptor, a hormone that controls circadian rhythms and melatonin pathways that are involved in the pathogenesis of T2D (40). A functional study of rs10830963 near *MTNR1B* has shown an increase in the risk of T2D through impaired insulin secretion, suggesting its role in beta cell dysfunction. The same study has shown that this variant is associated with increased expression of *MTNR1B* in islet cells and immunohistochemistry confirmed its colocalization in beta cells (41). In addition, a study in the Chinese population has shown that the T2D patients carrying the G-risk allele of rs10830963 have decreased efficacy towards nateglinide treatment (42). As observed in HOMA2-IR, a novel variant rs61552983 at 4q34.3 locus near *VEGFC* has reached a significant genome-wide association with beta cell function (HOMA2-%B) but only without adjusting for BMI. Although this SNP has neither been previously associated with beta cell function nor with BMI, SNPs within 250 kb window of the SNP have been reported to be associated with BMI (43). The observed association in non-BMI adjusted model (model A) could be partly driven by BMI and could be due to potential pleiotropy of the locus in BMI, IR and beta cell function. This finding underscores the intersecting pathophysiology of obesity and IR and highlights the need to correct for BMI in studies of IR. *VEGFC* is a member of the platelet-derived growth factor family, which encodes a protein that promotes angiogenesis and endothelial cell growth. A meta-analysis study has shown that the expression of *VEGFC* was significantly higher in group of obese individuals compared to non-obese (44). While the C-type *VEGF* has not been well studied in the context of beta cell function, its isoform, the A-type (*VEGFA*) has been shown to play a vital role in beta cell development and differentiation (45). Lastly, the rs61552983 showed borderline evidence of heterogeneity (*Q* = 0.031) which could be attributed to differences in the effect size of rs61552983 between discovery and replication cohorts. The effect size (beta) was higher in discovery (-0.346) than in replication (-0.157). However, the direction of effect size in both cohorts was the same. Altogether, we have shown that *VEGFC* has a potential role in IR and beta cell function which deserve further exploration.

PGS have attracted the attention of researchers and clinicians as predictors for diseases and complex traits. While the clinical utility of PGS is still limited, accumulating evidence supports their future use in clinics. We developed 6 PGS using the clumping and thresholding method and identified 3 PGS that best predict IR in the Qatari population. Moreover, our top Q-PGS was based on 1,390 variants (Q-PGS4) and it outperformed PGS003470 that was based on 775,999 variants (22). It is important to note that while PGS003470 was developed for IR from 37,037 European individuals, its performance in predicting IR has not been tested before.

The improved performance of our PGS compared to PGS003470 could be attributed to differences in LD patterns, and/or differences in allele frequencies of the variants between populations (11). However, potential of overfitting cannot be fully excluded as our PGS was derived and tested on the same population. Overall, our findings underscore the importance of accounting for population-specific genetic architecture, suggesting that even smaller sets of variants tailored to the Qatari population can yield higher predictive accuracy. These findings have significant implications for precision medicine, highlighting the potential of population specific PGS in identifying individuals at risk of IR within distinct populations, thereby enabling more targeted interventions and personalized treatment strategies. Our Q-PGS therefore has potential application to be used as a tool for predicting IR and identifying individuals at higher risk of IR, which is a strong risk factor of diabetes, metabolic syndrome, hypertension, obesity, fatty liver disease, cardiovascular disease and other IR-related abnormalities (46).

It is noteworthy that we could not detect many genome-wide signals partly due to the limited sample size to detect associations for variants with small effect size. Our study was only sufficiently powered to detect variants with effect size (beta) ≥ 0.175 and many previously reported loci have smaller effect size. Moreover, as the pioneering GWAS on HOMA2, we could not compare our findings with data from other populations for direct comparisons. However, our replication of most previously known loci identified from HOMA suggests that the two measures are broadly correlated and have shared genetic architecture. Further studies with larger sample size and functional analyses are warranted to further define the genetic architecture of IR that will ultimately assist in drug design and polygenic predictions for clinical translation.

## Data availability statement

GWAS summary statistics generated in this study have been deposited in the NHGRI-EBI Catalog of human genome-wide association studies and can be accessed through http://ftp.ebi.ac.uk/pub/databases/gwas/summary_statistics/GCST90428001-GCST90429000 under the accession codes GCST90428888, GCST90428889, GCST90428890 and GCST90428891.

## Ethics statement

The studies involving humans were approved by Institutional Review Boards of Qatar Biobank and Hamad Bin Khalifa University, Doha, Qatar. The studies were conducted in accordance with the local legislation and institutional requirements. The participants provided their written informed consent to participate in this study.

## Author contributions

UA: Data curation, Formal analysis, Investigation, Writing – original draft. U-KU: Formal analysis, Visualization, Writing – review & editing. ST: Investigation, Visualization, Writing – review & editing. AE: Data curation, Methodology, Writing – review & editing. YA-S: Investigation, Methodology, Writing – review & editing. AA-S: Funding acquisition, Writing – review & editing. KS: Funding acquisition, Writing – review & editing. OA: Conceptualization, Formal analysis, Funding acquisition, Investigation, Supervision, Writing – review & editing.
