# Supplementary material for: Genome-wide association study and polygenic score assessment of insulin resistance
Source: Front Endocrinol (Lausanne). 2024 Jun 13;15:1384103. doi: 10.3389/fendo.2024.1384103 (PMC11208314; doi:10.3389/fendo.2024.1384103)
Supplement: Supplementary file 1 [file DataSheet_1.pdf]

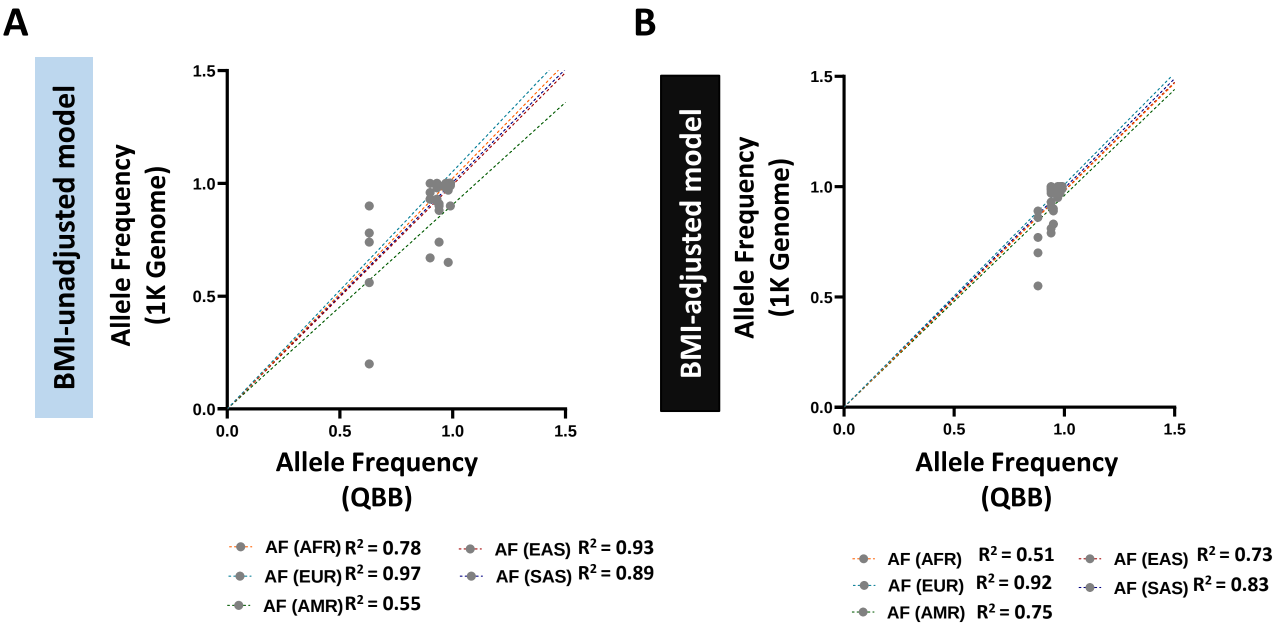

Supplementary Figure 1. Comparison of known HOMA-IR-associated loci between Qatar Biobank (QBB) and 1000 genome project (1K Genome). (A) Correlation of the allele frequency of the lead SNPs in QBB within ±250 kb of previously reported SNPs in model A and **(B)** model B between QBB and European (EUR), African (AFR), East Asian (EAS), South Asian (SAS) and Admixed American (AMR) ancestry subjects from the 1K Genome.

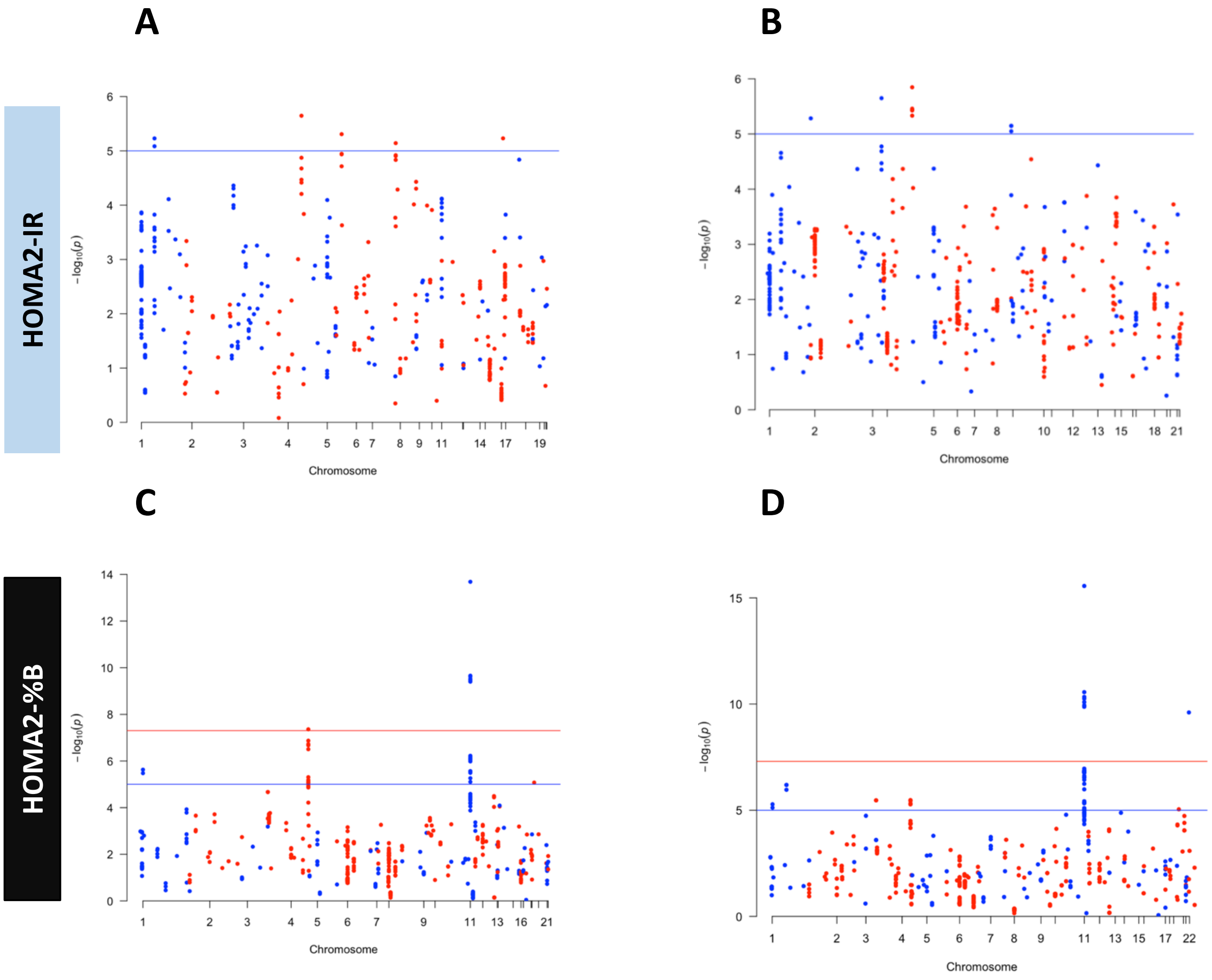

Supplementary Figure 2. Manhattan Plots for HOMA2-IR and HOMA2-%B from Meta-analyses. **(A)** Manhattan plot for HOMA2-IR in non-BMI adjusted (Model A) and **(B)** BMI-adjusted (Model B). **(C)** Manhattan plot for HOMA2-%B in non-BMI adjusted (Model A) and **(D)** BMI-adjusted (Model B). Manhattan plots represent the -log10 *P* (significance) on y-axis for SNPs represented on the x-axis based on their chromosomal position. The blue horizontal line represents suggestive evidence of association ($P<5\times10^{-5}$). The red horizontal line represents the genome-wide significance threshold ($P<5\times10^{-8}$). Meta-analyses was performed for SNPs that reached $P< 5 \times 10^{-5}$ in the discovery set and tested in the replication dataset.
